# HMGB1 prefers to interact with structural RNAs and regulates rRNA methylation modification and translation in HeLa cells

**DOI:** 10.1186/s12864-024-10204-6

**Published:** 2024-04-05

**Authors:** Meimei Liao, Jiarui Cao, Wen Chen, Mengwei Wang, Zhihui Jin, Jia Ye, Yijun Ren, Yaxun Wei, Yaqiang Xue, Dong Chen, Yi Zhang, Sen Chen

**Affiliations:** 1 Department of Ultrasound Imaging, Renmin Hospital of Wuhan University, Hubei Wuhan, People’s Republic of China; 2 Department of Orthopedics, Renmin Hospital of Wuhan University, Hubei Wuhan, People’s Republic of China; 3 Center for Genome Analysis, ABLife Inc., Optics Valley International Biomedical Park, East Lake High-Tech Development Zone, 388 Gaoxin 2Nd Road, Hubei Wuhan, 430075 China; 4 Laboratory for Genome Regulation and Human Health, ABLife Inc., Optics Valley International Biomedical Park, East Lake High-Tech Development Zone, 388 Gaoxin 2Nd Road, Hubei Wuhan, 430075 China

**Keywords:** HMGB1, iRIP-seq, 28S rRNA, snoRNAs, Structured RNA, Methylation modification, Translation, Cancer cells

## Abstract

**Background:**

High-mobility group B1 (HMGB1) is both a DNA binding nuclear factor modulating transcription and a crucial cytokine that mediates the response to both infectious and noninfectious inflammation such as autoimmunity, cancer, trauma, and ischemia reperfusion injury. HMGB1 has been proposed to control ribosome biogenesis, similar as the other members of a class of HMGB proteins.

**Results:**

Here, we report that HMGB1 selectively promotes transcription of genes involved in the regulation of transcription, osteoclast differentiation and apoptotic process. Improved RNA immunoprecipitation by UV cross-linking and deep sequencing (iRIP-seq) experiment revealed that HMGB1 selectively bound to mRNAs functioning not only in signal transduction and gene expression, but also in axon guidance, focal adhesion, and extracellular matrix organization. Importantly, HMGB1-bound reads were strongly enriched in specific structured RNAs, including the domain II of 28S rRNA, H/ACA box snoRNAs including snoRNA63 and scaRNAs. RTL-P experiment showed that overexpression of HMGB1 led to a decreased methylation modification of 28S rRNA at position Am2388, Cm2409, and Gm2411. We further showed that HMGB1 overexpression increased ribosome RNA expression levels and enhanced protein synthesis.

**Conclusion:**

Taken together, our results support a model in which HMGB1 binds to multiple RNA species in human cancer cells, which could at least partially contribute to HMGB1-modulated rRNA modification, protein synthesis function of ribosomes, and differential gene expression including rRNA genes. These findings provide additional mechanistic clues to HMGB1 functions in cancers and cell differentiation.

**Supplementary Information:**

The online version contains supplementary material available at 10.1186/s12864-024-10204-6.

## Background

High-mobility group B (HMGB) proteins are non-histone proteins which possess a unique DNA-binding domain, the HMG-box. There are four HMGBs in mammals, HMGB1-4 [1]. HMGB1 is the most important member of the high mobility group B proteins family. Early studies showed that HMGB1 is located in the nucleus and exerts important functions for chromatin remodeling acting as DNA-binding protein [2], DNA repair and gene expression [3]. In response to injury, infection, or other inflammatory stimuli, HMGB1 is secreted into extracellular space in the activated macrophages, mature dendritic cells (DCs) and natural killer (NK) cells to mediate inflammation and immune response [4–9]. HMGB1 activates cells through the differential engagement of multiple surface receptors including TLR2, TLR4, and RAGE [10].

HMGB1 is evolutionarily highly conserved, which contains two HMG-boxes domains and an acidic C-terminus [3]. HMG-box domains are involved in DNA binding and also interact with other proteins [11]. HMGB1 plays a plethora of cell regulatory functions, such as inflammation [12], mesenchymal stem cells differentiation [13] and cells death [14]. Besides, HMGB1 is an abundant chromatin protein that also affects osteoblastic differentiation. However, it is unclear how HMGB1 functions in this biological process [15]. HMGB1 is overexpressed in cancerous cells [16, 17], promoting FAK/PI3K/mTOR signaling cascade and enhancing cell proliferation and preventing apoptosis [18], although the opposite effects are also documented [19, 20].

Interestingly, two human HMGB proteins HMGB1 and UBF1 contain two or more HMG-box domains and bind to DNA with low sequence specificity [21]. It is emerging that this class of HMGB proteins control ribosome biogenesis [22]. UBF1 actively enhances rDNA transcription by interacting DNA polymerase I machinery and other transcription factors [23–27], A recent proteomic study has revealed the protein–protein interactions between HMGB1 and ribosomal proteins [17]. Nevertheless, it remains unclear how HMGB1 control ribosome biogenesis, and whether this control is related its regulation of cancer cell growth and apoptosis.

In vitro experiments using competitive electrophoretic mobility and circular dichroism binding assays revealed that HMGB1 binds to branched RNA structures including *E. coli* 5S rRNA and a group I intron ribozyme with high affinity [28]. Recent mRNA interactome studies have revealed that HMGB1 also binds to mRNAs [29, 30]. The recent study on the function of Hepatitis C virus (HCV) genome in recruiting host proteins has provided evidence that HMGB1 associates with HCV genome RNA to influence viral replication [31]. In fact, a number of transcription factors bind to both DNA and RNA species, suggesting a mechanism of cross-talking between the molecular processes occurring on DNA and RNA levels [32]. We therefore speculate that HMGB1 may exert some of its biological functions by its RNA binding activities in cancer cells.

To test this hypothesis, we overexpressed Flag-tagged HMGB1 (HMGB1-OE) in HeLa cells, and systematically explored the cellular influence by HMGB1-OE. Whole transcriptome sequencing analysis (RNA-seq) was performed to identify the molecular targets of HMGB1. We then utilized a Flag antibody to capture HMGB1-RNA interactome using the iRIP-seq approach, which allowed the determination of the HMGB1-bound RNA targets at whole transcriptome level in living cells. We next applied RTL-P experiment to study the potential impact of HMGB1-bound on the methylation modification of 28S rRNA. We further explored how HMGB1-OE modulates the protein synthesis process. Taken together, our results support a model in which HMGB1 binds to different RNA species with differential specificity in human cancer cells, which shall contribute to HMGB1-modulated rRNA modification, protein synthesis function of ribosomes, and differential gene expression including rRNA genes.

## Results

### RNA-seq profiling reveals the global transcription regulation associated with HMGB1 overexpression

**Fig. 1:**
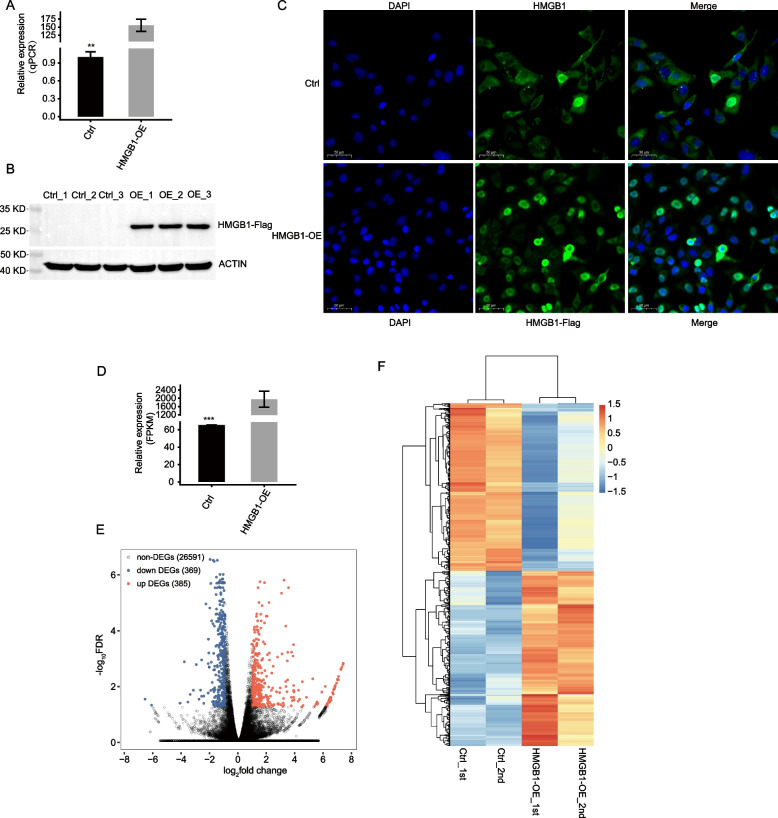
RNA-seq analysis of differential gene expression in response to HMGB1-OE. **A** HMGB1 expression in HeLa cells after transiently transfected with HMGB1 overexpression or control vector, as determined by RT-qPCR. **B** HMGB1 overexpression and control vector followed by Western blot using antibody against FLAG. Three replicates. **C** Immune fluorescence experiment showing the subcellular localization of HMGB1 and HMGB1-Flag in HeLa cells. **D** HMGB1 expression values quantified using RNA sequencing data. **E** Detection of the HMGB1 regulated genes on the volcano plots, up DEGs (FC ≥ 2, FDR < 0.05) are labeled red, whereas down DEGs (FC ≤ 0.5, FDR < 0.05) are labeled blue. (F) Hierarchical clustering of DEGs in control and overexpression cells (Expression values are log_2_-transformed and the median-centered by each gene)

To establish an effective cell model to study HMGB1 binding and regulatory targets in cancer cells, we overexpressed the Flag-tagged HMGB1 using a vector-based expression system in HeLa cells, and the Flag-only plasmid as control (Ctrl). The efficacy of Flag-HMGB1 overexpression (HMGB1-OE) was assessed by RT-qPCR and Western blot (Fig. 1A-B, Fig. S1A). Meanwhile, we performed immune fluorescence experiment to check the subcellular localization of HMGB1 in HeLa cells. Before overexpression, we found HMGB1 was mainly localized in nucleus (Fig. 1C, up panel). We also performed co-localization analysis between HMGB1 and Nucleolin, a nucleolus protein, and found their co-occurrence in HeLa cells (Fig. S1B). After overexpression, we detected the translocation to cytoplasm of HMGB1-Flag (Fig. 1C, bottom panel), indicating that HMGB1-OE altered the subcellular localization of HMGB1 protein, which could in turn modulate the functions of HMGB1 in HeLa cells. To assess whether HMGB1 modulates transcription as previously reported, we performed RNA-seq analysis on the HeLa cells between HMGB1-OE *vs.* Ctrl. After removing adaptor sequences and low-quality sequencing reads, we obtained a total of 58.1 ± 2.2 million high-quality reads from each sample (Table S1). RNA-seq yielded robust expression for 27,312 genes (Table S2). Effective overexpression of HMGB1 was further confirmed in parallel RNA-seq analysis (Fig. 1D). Using software DESeq2 [33], a total of 754 differentially expressed genes (DEGs) (Fold Change ≥ 2 or ≤ 0.5, FDR < 0.05) were obtained between HMGB1-OE *vs.* Ctrl cells, including 385 up-regulated and 369 down-regulated genes by HMGB1-OE, respectively (Table S3), which agrees with the known transcription regulator function of HMGB1 [5, 11, 34]. A volcano plot was constructed to display genes whose expression was significantly changed in response to HMGB1 expression (Fig. 1E). Heatmap analysis of the expression patterns of the DEGs in RNA-seq samples showed a high consistency of the HMGB1-OE mediated transcription in both data sets (Fig. 1F). At the same time, we performed quantitative proteomics experiment for HMGB1-OE and Ctrl samples. Sample correlation analysis revealed the 0.5 correlation coefficient between the RNA-seq and proteomics samples (Fig. S1C), indicating that the translation process of a large part of genes may be independent of the transcription regulation.

Meanwhile, we also knocked down HMGB1 (HMGB1-KD) in HeLa cells and performed RNA-seq to explore its effect on transcriptome profile (Fig. S1D). DEG analysis revealed 64 up and 139 down DEGs (Fig. S1E-F), which were much less than that by HMGB1-OE. We then overlapped the two DEG sets between HMGB1-OE and HMGB1-KD, and found very few genes, including 12 up and 1 down DEGs in HMGB1-OE, were consistently overlapped in these two datasets (Fig. S1G). These results indicate that HMGB1-OE has more pronounced regulation on transcriptome than HMGB1-KD in HeLa cells, thus we used the HMGB1-OE RNA-seq data for following analysis.

### HMGB1 selectively regulates the expression of genes involved in transcriptional regulation pathways

**Fig. 2:**
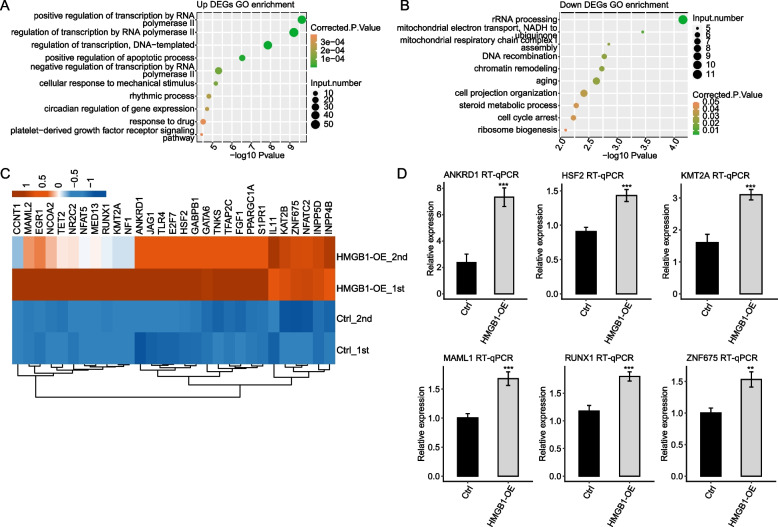
The significantly overrepresented pathways identified by enrichment analysis of differentially expressed genes. **A** and **B** The top 10 representative GO biological process terms of HMGB1-regulated genes. X-axis represents the base 10 logarithm of the enrichment *p*-value, y-axis represents the term of enriched GO pathways. Up means up-regulated genes in OE-HMGB1 cells, down means down-regulated genes in OE-HMGB1 cells. **C** Heatmap of transcription factor genes in control and HMGB1-OE cells. In the heatmap, colors ranged from blue to red, correspond to low-to-high expression level in cells. **D** Validation of some DEGs by identical RT-qPCR assay

To correlate the HMGB1-regulated gene expression and biological functions, we subjected all 754 DEGs to GO annotation (Table S4 and Table S5). In the biological processes (BPs) of GO analysis, the up-regulated genes in the HMGB1-OE samples were highly enriched in the positive regulation of transcription from RNA polymerase II promoter, negative regulation of osteoclast differentiation, positive regulation of apoptotic process, and DNA-dependent transcription (Fig. 2A). The down-regulated genes were also enriched in the DNA-dependent transcription, and rRNA processing (Fig. 2B).

HMGB1-upregulated genes related to transcription from RNA polymerase II promoter included *ANKRD1, KMT2A, CSRNP1, IL11, GABPB1, E2F7, RUNX1, TNKS, TFAP2C, JAG1, CCNT1, NFAT5, MED13, S1PR1, NR2C2, TLR4, MAML2, GATA6, NFATC2,* and *HSF2*. Notably, HMGB1-OE significantly promoted genes related to osteoclast differentiation processing, such as *ZNF675, TLR4* and *NF1* (Fig. 2C). *TLR4* is known to facilitate osteoblastic differentiation of mesenchymal stem cell [15]. In addition to those involved in transcription, some genes related to cell apoptosis such as *DAPK1* and *STK17A* were also affected by HMGB1.

KEGG enrichment analysis was also performed (Table S6). Multiple DEGs were enriched in cancer pathway, transcriptional misregulation in cancer pathway, and signaling pathway. Representative genes from RNA polymerase II promoter transcription (*ANKRD1, KMT2A, IL11, RUNX1, JAG1, S1PR1, TLR4, MAML2, NFATC2, and HSF2*) and osteoclast differentiation (*ZNF675* and *NF1*) were selected for RT-qPCR validation of their mRNA levels in HMGB1-OE and Ctrl cells, showing high consistency with the RNA-seq analysis (Fig. 2D).

### iRIP-seq analysis of HMGB1-bound RNA targets in HeLa cells

**Fig. 3:**
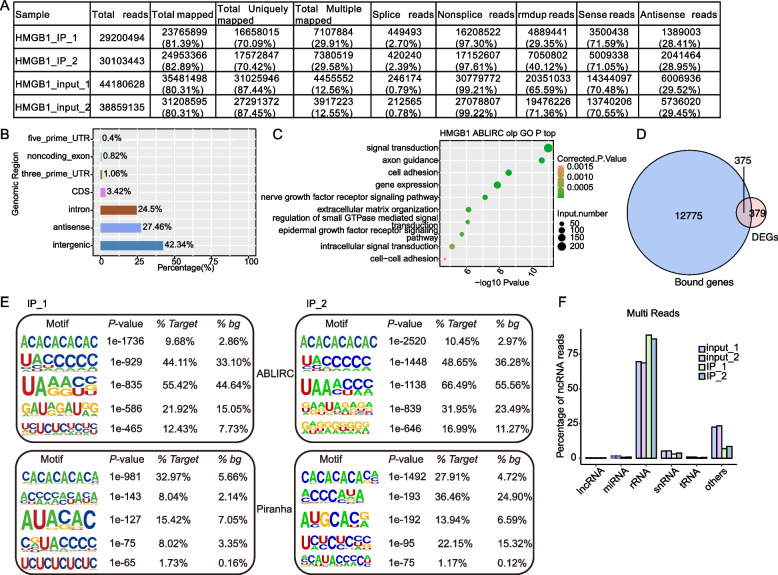
iRIP-seq of HMGB1 bound-RNAs in HeLa cells.** A** Table summarizing the number of total mapped reads to the human genome for the iRIP-seq duplicates. **B** Genomic distribution of HMGB1-bound peaks called by ABLIRC algorithm. **C** The top 10 representative GO biological process terms of HMGB1-regulated genes overlap with HMGB1-bound genes. X-axis represents the base 10 logarithm of the enrichment *p*-value, y-axis represents the term of enriched GO pathways. **D** Venn diagram showing the overlapped genes between DEGs by HMGB1-OE and HMGB1-bound genes. **E** Extracted HMGB1 peaks motifs using ABLIRC or Piranha. **F** Percentage of iRIP-seq reads that blast to the Rfam database: long intergenic ncRNA (linRNA), microRNA (miRNA), ribosomal RNA (rRNA), 5S ribosomal RNA (5S rRNA), 5.8S ribosomal RNA (5.8S rRNA), small nuclear RNA (snRNA), transfer RNA (tRNA) or other ncRNA relative to the total number of ncRNA. Red and dark blue column represent IP1 and IP2, respectively

The above results established a functional platform for further analysis of the ectopically expressed HMGB1-bound RNA targets in HeLa cells. In order to assess the specificity of HMGB1 pull-downs, we set Input as the control of immunoprecipitation. Two replicate libraries of HMGB1 iRIP-seq (IP_1 and IP_2) were sequenced. After removing adaptor sequences and low-quality reads, a total of 29.7 and 30.8 million reads were obtained for IP_1 and IP_2, respectively. A total of 44.8 and 39.4 million reads were recovered from the two Input samples (Table S7). When these reads were mapped onto the human GRCh38 genome using TopHat2 [35], we obtained 14.95 and 15.92 million uniquely aligned reads, respectively. Notably, the fractions of multiple aligned reads in IP_1 (39.75%) and IP_2 (39.61%) were higher than the Input 1 (23.16%) and Input 2 (23.29%) (Fig. 3A).

To identify HMGB1 binding sites in protein-coding genes, we used uniquely mapped reads from the IP and Input samples. Here, we adopted two different software ABLIRC [36] and Piranha [37] to recover the HMGB1 binding sites (peaks) from the iRIP-seq reads (Table S8). We then focused on the confident peaks and associated genes identified in both replicates, and identified 13,150 HMGB1-bound genes. The peaks distribution showed a broad range of binding sites to intergenic regions (42.34%), antisense (27.46%), introns (24.5%), coding sequences (3.42%), 3’-UTR (1.06%), 5’-UTR (0.4%), and noncoding gene exons (0.82%) (Fig. 3B).

Then we performed the GO and KEGG enrichment analysis to analyze the BPs enriched by HMGB1-bound confident genes (Table S9 and Table S10). The top 10 enriched BPs included not only “signal transduction”, “gene expression” and “regulation of small GTPase mediated signal transduction”, but also “axon guidance”, “cell adhesion” and “extracellular matrix organization (Fig. 3C and Fig. S2A-B). Interestingly, in the HMGB1_IP samples, out of the HMGB1-bound signal transduction pathway mRNAs, several were found to encode proteins involved in cell growth and ribosome biogenesis, including cell growth factor (e.g. *GDF15, CDKN1A, VEGF, FGF7, FGFR2,* and *EGFR* mRNA), translation initiation factor (e.g. *EIF4E, EIF4EBP1*, *EIF4A2*), ribosome synthesis ( e.g. *mTOR, RPS2, RPL10*) and cell cycle regulator (e.g. *CCND2*).We elucidated the correlation between HMGB1 binding of mRNAs and differential gene expression. Notably, 375 DEGs were overlapped with HMGB1-bound mRNAs (Fig. 3D). Furthermore, we searched for the motifs in HMGB1_IP_1 and HMGB1_IP_2 binding peaks respectively using Homer software [38]. (CA)-repeat motif was strongly enriched in both ABLIRC and Piranha-peaks (Fig. 3E). At the same time, we noticed that one study systematically identified the chromatin binding sites and interacting transcripts of HMBG1 in IMR90 cells by ChIP-seq and sCLIP-seq, respectively [39]. Then we analyzed the overlapped genes between our iRIP-seq results and the ChIP-seq and sCLIP-seq results. After comparison, we found most of the transcripts from sCLIP-seq dataset were also included in our iRIP-seq dataset (Fig. S2C, *p*-value = 9.87e-11, hypergeometric test). While there were few overlapped genes between ChIP-seq dataset and our iRIP-seq dataset (Fig. S2D, *p*-value = 1, hypergeometric test), indicating that HMGB1-bound transcripts showed consistency in different cells. We then analyzed the enriched functions for the 288 overlapped genes between sCLIP-seq and iRIP-seq, and found they were enriched in cell adhesion, extracellular matrix, and cell growth associated pathways (Fig. S2E).

As the multiple aligned reads were higher in HMGB1 iRIP-seq reads compared with the input controls (Fig. 3A), we further studied where these reads came from. The multiple aligned reads were further mapped to those collected and annotated in Rfam (http://xfam.org/) database, which revealed 56.4% and 56.1% of the sequences mapping to Rfam database from two sets of IP samples (Table S11). Clearly, when compared with those in input controls, the fractions of HMGB1 iRIP-seq mapped onto rRNA genes were significantly higher (Fig. 3F).

### HMGB1 specifically binds to domain II of 28S rRNAs

As shown in Fig. 3F, we showed that a large fraction of HMGB1-bound reads was derived from rRNAs. We then further studied the characteristics of HMGB1 binding of rRNAs. rRNAs are encoded by the 5S and 45S ribosomal DNA (rDNA) genes of eukaryotes [40]. The 5S rDNA gene resides on chromosome 1 and encodes the 5S rRNA, whereas the 45S rDNA gene resides on five human acrocentric chromosomes and encodes the 18S, 5.8S and 28S rRNA components of the ribosome [41]. The human 45S rDNA (GenBank reference number U13369) contains the 18S, 5.8S and 28S rRNA encoding regions and the promoter, internal transcribed spacer (ITS) as well as external transcribed spacer (ETS) regions, and an intergenic segment (IGS) [42].

**Fig. 4:**
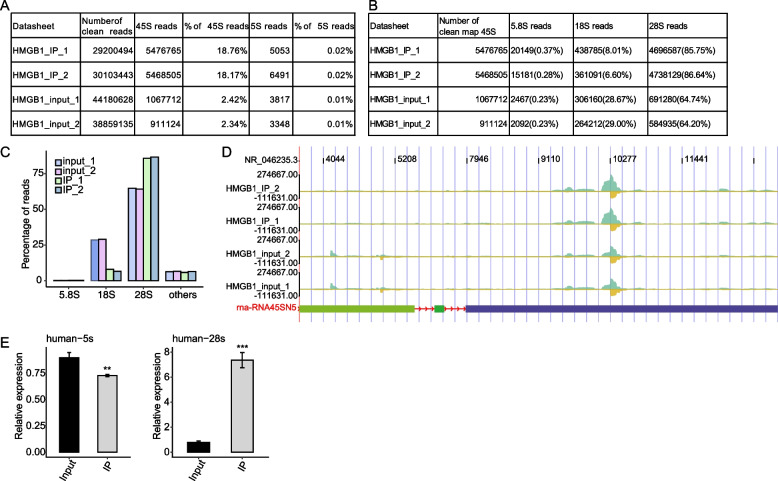
Distribution of ribosomal RNA reads in HMGB1 iRIP-seq. **A** Summary table for the analyzed iRIP-seq reads mapped to the 45S and 5S rDNAs from two IP and Input samples. **B** Summary table for the iRIP-seq reads mapped to the 5.8S, 18S, and 28S rDNAs. **C** Bar plot for the iRIP-seq reads mapped to the 5.8S, 18S, and 28S rDNAs. **D** Reads distribution of the human 28S rRNA. The diagram reflected the abundant binding region of the HMGB1 in 28S rRNA domain II and modification location. **E** RIP-qPCR validation of HMGB1-bound rRNA

We extracted the sequences of 5S and 45 S rDNA genes [43], and mapped all IP reads one these sequences. A total of 6.4 and 6.6 million reads were mapped onto the 45S rDNA from two sets of IP samples, respectively, and only a few thousand reads were mapped onto 5S rDNA (Fig. 4A). They represent 21.54% and 21.5% reads from two sets of IP samples, while only 3.58–3.68% input reads were mapped onto the 45S rDNA sequence (Fig. 4A). Among the reads mapped onto 45S rRNA, 4,697,522 (73.43%) and 4,742,867 (71.54%) reads mapped to the 28S rDNA from the two IP samples, which were higher than the input controls (41.52–42.02%). In contrast, the fractions of 18S rRNA reads in input samples were higher than the HMGB1 IP samples (Fig. 4B-C). Mapping of the HMGB1-bound reads density onto the secondary structure of 28S rRNA revealed that the highest hit density was exclusively resided in the 28S rRNA Domain II region, as well as in a very small density of domain V (Fig. 4D). To confirm whether HMGB1 directly interacts with 28S rRNA, RNA isolated from the HMGB1-complex by RNA IP was analyzed by RT-qPCR. These results indicated that HMGB1 co-precipitated with 28S rRNA but showed little binding to 18S rRNA, 5S and 5.8S rRNA (Fig. 4C and E).

### HMGB1 selectively binds to snoRNAs, particularly H/ACA box type, and scaRNAs

**Fig. 5:**
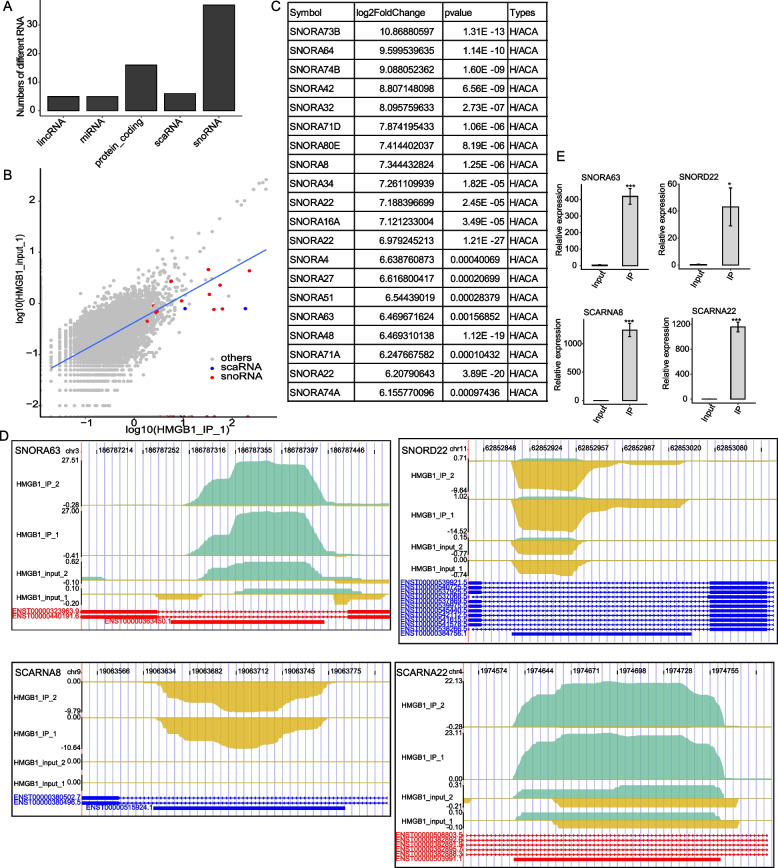
HMGB1 binds snoRNAs and scaRNAs with high affinity and affect rRNA modification. **A** Bar plot represent for the indicated Top-100-bound RNA biotypes in the IP and Input samples. (A cut off of IP/input fold change value ≥ 2 and *p*-value < 0.05). **B** Scatter plot showing comparison between expression profiles of IP (x axis) and Input (y axis) (Expression values are log10-transformed). snoRNAs are colored in red, scaRNA are colored in green, others are in grey. **C** The top 20 enriched snoRNA enriched in HMGB1 IP sample (*p*-value < 0.05). **D** The distribution of reads across the whole region in the snoRA63 and snoRD22 genomic location and RIP-qPCR validation of HMGB1-bound snoRNA. And validation of HMGB1-bound scaRNA by RIP-qPCR. **E** Bar plot showing the uvRIP-qPCR results for four snoRNAs/scaRNAs that were bound by HMGB1

We next explored whether small structured RNAs were selectively bound by HMGB1. As small structured RNAs were normally expressed at high levels and single-copied, we used uniquely mapped reads from the IP and Input samples to analyze the small RNAs bound with HMGB1. We extracted the top 100 abundant RNAs from the uniquely mapped results, showing that most were small structured RNAs, including snoRNAs (37 in IP sample), miRNAs (2 in IP sample) and scaRNAs (5 in IP samples) (Fig. 5A). A scatter plot shows the uniquely mapped IP read density (FPKM) of IP and input samples in all hit genes, with those of the top 100 abundant RNAs being highlighted with red colors (Fig. 5B and Table S12). We demonstrated that 37 snoRNAs and 5 scaRNAs among the top 100 abundant RNAs were enriched in HMGB1 IP samples compare with the Input samples, using a cut-off of IP/input fold change value ≥ 2 and *p*-value < 0.05. In contrast, most of the other type of 100 top abundant RNAs were not enriched in HMGB1 IP samples (Fig. 5C and Table S12). We further analyzed the enrichment of all expressed snoRNAs in HMGB1 IP samples, demonstrating 28.2% (49/174) was significantly enriched. Among the top 20 most enriched snoRNAs in HMGB1 IP samples, all of them belonged to H/ACA box type (Fig. 5C).

These HMGB1-bound snoRNAs include snoRA63, snoRA73b and snoRD22, scaRNA22, scaRNA8 and scaRNA13 (Fig. 5C-D and Fig. S3A). To validate the direct binding events identified in data analysis, these mentioned HMGB1-bound snoRNA and scaRNA were analyzed by uvRIP-qPCR (Fig. 5E and Fig. S3B). The results from uvRIP-qPCR and iRIP-seq analysis were highly consistent, suggesting the confidence of HMGB1 binding of snoRNAs and scaRNAs.

### HMGB1 overexpression decreased the methylation modification of 28S rRNA

**Fig. 6:**
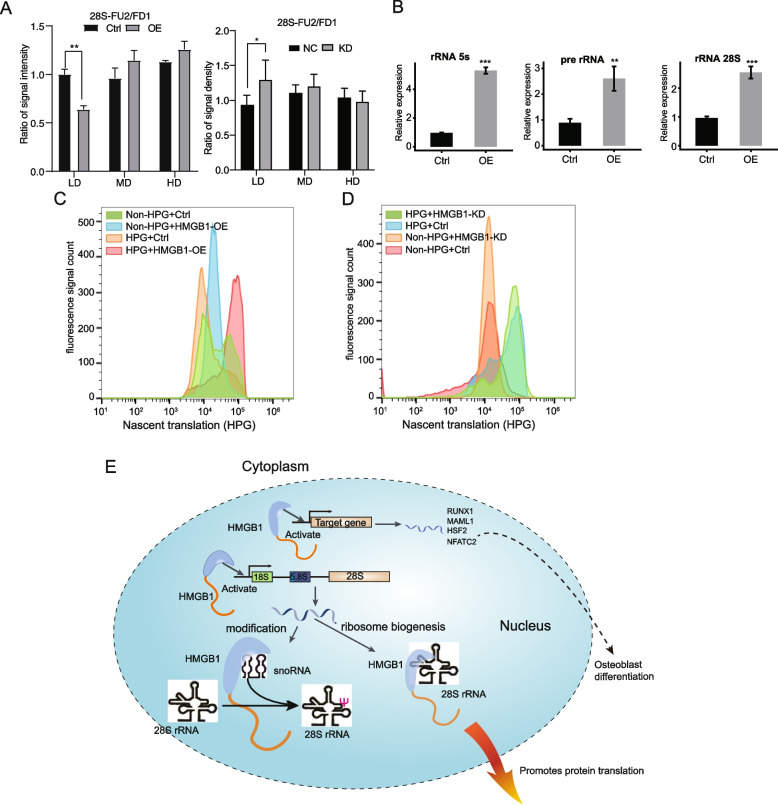
HMGB1 overexpression decreased the methylation modification of 28S rRNA and promote rRNA transcription and protein synthesis. **A** The 28S-FU2-RT/FD1-RT signal intensity ration detected by RT-qPCR in HMGB1-OE (left panel) and HMGB1-KD (right panel samples). *n* = 3 biological replicates. LD: low dNTP; MD: middle dNTP; HD: high dNTP. **B** RT-qPCR showing the increase of rRNA levels after HMGB1 overexpression. *n* = 3 biological replicates. **C** Representative histogram showing the HPG fluorescent staining signal result in the protein synthesis activity in HMGB1-OE and Ctrl cells. Three replicates. **D** Representative histogram showing the HPG fluorescent staining signal result in the protein synthesis activity in HMGB1-KD and Ctrl cells. Three replicates. **E** Diagram depicting a simplified model of HMGB1 influence on ribosome modification, rRNA expression and protein synthesis

The primary function of snoRNAs is guiding chemical modifications of other RNAs, mainly ribosomal RNAs, transfer RNAs and small nuclear RNAs. The C/D box snoRNAs are associated with methylation, and the H/ACA box snoRNAs with pseudouridylation [44]. To test the consequences of HMGB1-bound snoRNAs on rRNA modifications, we obtained total RNA from the Ctrl and HMGB1-OE HeLa cells, and then applied RTL-P assay to validate specific 2’-O-methylation sites in 28S rRNA. We examined the modified methylation level at positions 2388, 2409, and 2411 of the 28S rRNA, which are the modification sites and located within HMGB1-bound region. It is demonstrated that the read-through PCR product level at low dNTP was significantly decreased upon HMGB1-OE and increased in HMGB1-KD at low concentration (Fig. 6A), indicating an increased methylation modification at this site in HMGB1-OE samples.

### HMGB1-OE increases rRNA level and protein synthesis

To our surprise, HMGB1-OE significantly increases the steady-state level of both 28S rRNA and 5S rRNA, as well as the pre-rRNA in the 5’ external transcribed spacer (5’ETS) for 28S rRNA (Fig. 6B). This result could be explained by a model in which HMGB1-OE may increase the transcription of both 5S and 45S rRNA genes or stabilize their RNA levels, although we could not exclude the other possibility.

Both HMGB1-snoRNA binding associated regulation of rRNA modification and HMGB1-promoted rRNA steady-state levels suggest that HMGB1 may regulate protein synthesis. To test this hypothesis, we incubated Ctrl and HMGB1-OE cells with L-homopropargylglycine (HPG), an analog of methionine that can be detected with Click-iT™ HPG Alexa Fluor™ 488 Protein Synthesis Assay Kit (Thermos Fisher Scientific, C10428). The Ctrl Flag plasmid and HMGB1-OE Flag plasmid both harbored the GFP expressing cassette. Consistently, similar population of cells were detected with the fluorescence signal in the Ctrl and HMGB1-OE Flag samples, even in the absence of HPG (Fig. 6C). In the presence of HPG, a much large population of HMGB1-OE Flag cells (red peak) was detected with the fluorescence signal, while the population of fluorescence cells became even smaller in the Ctrl Flag expressing samples (brown peak) (Fig. 6C). Meanwhile, we performed the same experiment on HMGB1-KD and corresponding Ctrl samples. The results demonstrated that, in the presence of HPG, HMGB1-KD (green peak) did not obviously change the population of fluorescence cells comparing with the Ctrl samples (blue peak) (Fig. 6D). Taken together, these results support the conclusion that HMGB1-OE could regulate protein synthesis. Thus, we finally summarized our discovery of the functions of HMGB1 in transcription, RNA modification, and protein translation regulation in HeLa cells (Fig. 6E).

## Discussion

HMGB1 is not only a nuclear factor regulating chromatin remodeling or DNA repair [45], and gene expression [11], but also a secreted factor eliciting inflammatory response in autoimmunity, cancer, trauma, and ischemia reperfusion injury [5, 10]. HMGB1 binds to DNA with low specificity and belongs to a family of HMGBs regulating ribosome biogenesis, although this regulatory function has not been determined for HMGB1 [22]. The capability of HMGB1 interacting with structured RNA in vitro and with mRNA in cells is emerging [28–30]. In this study, we have confirmed the function of HMGB1 in mediating transcriptional regulation in HeLa cells, and found that HMGB1 selectively regulates the expression of transcription factors (TFs), particularly those involved in osteoclast differentiation. We provided evidence for HMGB1 binding of mRNA/pre-mRNAs enriched in KEGG pathways of focal adhesion, regulation of actin cytoskeleton and axon guidance, and pathways in cancer and PI3K-Akt signaling pathway. HMGB1-bound RNA reads were strongly enriched in specific structured RNAs, including the domain II of 28S rRNA, snoRNAs and scaRNAs. RTL-P experiment validated the potential impact of HMGB1 on decreasing the methylation modification of 28S rRNA at the target sites Am2388, Cm2409, and Gm2411. We further showed that HMGB1 increased the levels of 5S and 45S rRNAs, regardless of its RNA binding activity. Our results support a model in which HMGB1-regulated transcription together with HMGB1-28S rRNA and HMGB1-snoRNA interactions and collectively contribute to rRNA modification, ribosome biogenesis and protein synthesis in HeLa cells (Fig. 6E).

Consistent with the previously reported function of HMGB1 in regulating transcription from RNA polymerase II promoters [17, 23–27], we found that the levels of large number of mRNAs were under HMGB1 regulation in HeLa cells. HMGB1 selectively promoted the expression of genes involved in positive regulation of transcription from RNA polymerase II promoter, indicating an additional feed forward mechanism for HMGB1-mediated transcriptional activation. HMGB1-promoted genes are highly enriched in osteoclast differentiation, consistent with the reported function of HMGB1 in regulating osteoclast genesis in a manner dependent on RAGE [46]. Among these HMGB1-promoted TFs and co-factors, several are involved in cell differentiation and osteoclast development. KMT2A is an important epigenetic regulator during cell differentiation [47]. NFATC2 is a transcription factor and plays an important role in osteoclastogenesis [48]. HSF2 is a TF and is activated during cell differentiation [49]. TLR4 is involved in HMGB1-induced osteoblast migration [50]. MAML1, as a homologue of MAML2, can promote RUNX2 activity and regulate bone development [51]. These findings together indicate the additional regulatory cues to support osteoclastogenesis, which is illustrated in Fig. 6E.

HMGBs harboring two HMG-box domains bind to DNA with low specificity and encodes different functions than those harboring one HMG-box domain [22]. Hmo1, the yeast homologue of HMGB1, binds to the promoter and gene body region of 45S rDNA operon, creating DNA loops to stabilize the open chromatin, and regulates its transcription by RNA Pol I [52]. The other members of this HMGB family such as yeast Ixr1 and human UBF1, also controls RNA Pol I transcription from rDNA clusters [22]. In this study, we reported that HMGB1 promotes the expression of not only its bound 28S rRNA, but also the unbound 5S rRNA, and the spacer region in the 43S rDNA cluster, highly supporting the hypothesis that HMGB1 promotes transcription from rDNA promoters. This activation can be achieved by a direct binding of HMGB1 or via other TFs. Among the HMGB1-promoted TFs, RUNX1 belongs to the runt domain TFs that directly regulates the transcription of ribosome protein genes [53]. Meanwhile, we found that a large number of DEGs were overlapped with HMGB1-bound transcripts from iRIP-seq data, indicating that HMGB1 may also regulate expression level by binding to their transcripts and affecting their stability in HeLa cells. The conclusion that HMGB1 binds to transcripts was supported by the integrated analysis with HMGB1 sCLIP-seq data but not ChIP-seq data from another study [39], indicating HMGB1 could independently bind to DNA and RNA in cells. Another explanation is that HMGB1 has the ability to regulate different classes of genes by distinguishably binding to their DNA promoters or RNA transcripts, including mRNAs, rRNAs, snoRNAs, and scaRNAs that were discussed in following part. Further studies are necessary to clarify the dominant regulatory manner for HMGB1 on gene expression.

From the iRIP-seq result, we demonstrated that HMGB1 binds to 28S rRNA mapped to residues 1776–2242 in Domain II of 28S rRNA with high affinity. This region contains high G-C content, an expansion segment and the A-site finger domain [54]. Expansion segment 15 ( ES15^L^), a large segment in 28S rRNA, is substantially exposed at the ribosome surface and could be available for association with mRNAs as well as with non-ribosomal proteins [55]. The A-site finger is formed by a long helix (Helix 38) in domain II and is conserved in three kingdoms of life [56]. In eukaryotic cells, RNA modification is clustering in domain II of 28S rRNA [57], which may affect the rate of protein synthesis [58]. Although it remains to be determined how HMGB1 specifically recognizes and binds to this domain, it is likely that the long double helical structure and surface location of ES15^L^ participates in HMGB1 interaction. Given the importance of domain II in the ribosome assembly and translation activity, HMGB1 selective binding of this domain leads to a hypothesis that HMGB1 regulates ribosome biogenesis and translation probably by affecting its structure, which needs to be further verified using additional technologies and experiments.

The snoRNAs are central to the formation, trafficking and function of snoRNP catalyzing ribosomal RNA modifications, among which snoRNAs act as a guide to identify the modification sites via base pairing with a targeting region [59, 60]. Our iRIP-seq analysis revealed that snoRNAs, particularly H/ACA box snoRA63, snoRA73b and C/D Box snoRD22, are strongly selected by HMGB1. We further showed that HMGB1 decreased the methylation level at Am2388, Cm2409, and Gm2411 sites at 28S rRNA. These results together agree with a speculation that HMGB1 binding of snoRNAs may enhance its function in guiding the methylation modification of 28S rRNA. Considering that rRNA modifications play an important biological role in ribosome biogenesis, improving the fidelity of protein biosynthesis and rRNA stability [61], the formation of HMGB1-snoRNAs complex may improve protein synthesis in cancer cells by regulating methylation modifications of ribosome RNAs. Consistent with this prediction, we showed that protein synthesis activity in HeLa cells was distinctly promoted by HMGB1 overexpression.

Additionally, HMGB1 also selectively binds to the C/D box snoRNAs and small Cajal body-specific RNAs (scaRNAs). The later are a class of small nucleolar RNAs (snoRNAs) that specifically localize to the Cajal body, a nuclear organelle (cellular sub-organelle) involved in the biogenesis of small nuclear ribonucleoproteins (snRNPs), and guide the modification (methylation or pseudouridylation) of spliceosomal RNAs U1, U2, U4, U5 and U12 [62, 63]. We speculate that HMGB1 may affect spliceosome biogenesis and alternative splicing as well, which requires further study in the future. ScaRNAs are important regulators of development, cancers, and other diseases. ScaRNA2 is an important player of the DNA damage [64]. ScaRNA22 that maps to the intron of the WHSC1 gene is known to be involved in cell proliferation and stress response in multiple myelomas [65]. Therefore, HMGB1 interaction with scaRNAs proposes additional mechanism for the HMGB1 regulatory function in cancer, autoimmunity, as well as in many development-related processes. Based on the above discussion, we propose that besides the canonical regulatory function of HMGB1 on gene expression, HMGB1 could also synergistically bind to functional RNAs and finally influence cellular behaviors, indicating the multi-functional roles and yielding a novel regulatory layer of HMGB1 in cells. Due to the limited exploration depth of this study, further experiments should be performed to clarify the dominant regulatory manner of HMGB1 in particular cells and conditions.

## Conclusions

In conclusion, this study has demonstrated that HMGB1 regulates the transcription of hundreds of genes, several TFs that may contribute to osteoclast differentiation and osteoclastogenesis, and of RUNX1 contributing to ribosome biogenesis. We have shown that HMGB1 binds to mRNA/pre-mRNAs enriched for neuron cell related functions. More importantly, HMGB1 strongly selects domain II of 28S rRNA and H/ACA box snoRNAs, boxC/D snoRNAs, and scaRNAs as its targets. Furthermore, HMGB1 regulates the methylation level of HMGB1-bound snoRNA target, and enhances the protein synthesis. Taken together, this study proposes that HMGB1-structred RNA interaction in cancer cells is highly selective for rRNA modification, which may be integrated with HMGB1-promoted rRNA levels to increase protein synthesis in cancer cells. The findings provide additional mechanistic clues to HMGB1 functions in cancers, osteoclastogenesis, and likely in neuronal functions as well.

## Materials and methods

### Cell culture and transfection

HeLa cells (CCTCC@GDC0009) were obtained from The China Center for Type Culture Collection, and were then cultured under standard conditions with Dulbecco’s modified Eagle’s medium (DMEM) with 10% fetal bovine serum (FBS), 100 µg/mL streptomycin, and 100 U/mL penicillin. For HMGB1-OE, the coding sequence of HMGB1 was cloned into plasmid pIRES-hrGFP-1a containing a 3X Flag tag. For HMGB1 knockdown, the siRNA duplexes were purchased from Gemma (Suzhou, China). The siRNAs for HMGB1 and NC were GGAGAGAUGUGGAAUAACATT and UGUUAUUCCACAUCUCUCCTT, respectively. Transfection of the plasmids into HeLa cells was performed using lipofectAMINE 2000 (Invitrogen, Carlsbad, CA, USA) according to the manufacturer's protocol. Transfected cells were harvested after 48 h for following analysis.

### Assessment of HMGB1 expression by RT-qPCR and western blot analysis

The cDNA synthesis was done by standard procedures and real time PCR was performed on the Bio-Rad S1000 with Bestar SYBR Green RT-PCR Master Mix (DBI Bioscience). The concentration of each transcript was then normalized to GAPDH mRNA level using the 2^−ΔΔCT^ method [66]. Comparisons were performed with the paired Student’s *t*-test.

In brief, for the preparation of total cell lysates, the HeLa cells were lysed in RIPA buffer containing 50 mM Tris–HCl (pH 7.4), 150 mM NaCl, 1.0% deoxycholate, 1% Triton X-100, 1 mM EDTA and 0.1% SDS. The samples were centrifuged (12,000 rpm, 5 min) and the supernatants were further analyzed on a 10% SDS-PAGE gel and subsequently transferred to a PVDF membrane (Millipore). HMGB1 was detected using monoclonal Flag antibody (Sigma) and HMGB1 antibody (ab79823, Abcam) diluted in TBST (1:2,000), and Action (Abclonal) was used as a loading control (1:2,000).

### RNA-seq library preparation and sequencing

Total RNA was extracted by the TRIZOL (Ambion). The RNA was further purified with two phenol–chloroform treatments and then treated with RQ1 DNase (Promega) to remove DNA. The quality and quantity of the purified RNA were redetermined by measuring the absorbance at 260 nm/280 nm (A260/A280) using Smartspec Plus (Bio-Rad). The integrity of RNA was further verified by 1.5% agarose gel electrophoresis. For each sample, 1 μg of total RNA was used for RNA-seq library preparation by VAHTS Stranded mRNA-seq Library Prep Kit (Vazyme). Polyadenylated mRNAs were purified and fragmented, and then converted into double strand cDNA. After the step of end repair and a tailing, the DNAs were ligated to VAHTS RNA Adapters (Vazyme). Purified ligation products corresponding to 200–500 bp were digested with heat-labile UDG, and the single strand cDNA was amplified, purified, quantified, and stored at -80℃.

For high-throughput sequencing, the libraries were prepared following the manufacturer's instructions and applied to Illumina HiSeq X Ten system for 150 NT paired-end sequencing.

### Data processing and alignment

Raw reads containing more than 2-N bases were first discarded. Then adaptors and low-quality bases were trimmed from raw sequencing reads using FASTX-Toolkit (Version 0.0.13). The short reads less than 16 nt were also dropped. After that, clean reads were aligned to the GRCh38 genome by TopHat2 [35] allowing 4 mismatches. Uniquely mapped reads were used to calculate reads number and FPKM value (fragments per kilobase of transcript per million fragments mapped) [67] for each gene.

### Differentially expressed genes analysis

The DESeq2 [33] was utilized to screen out the differentially expressed genes (DEGs). A false discovery rate (FDR) < 0.05 and fold change ≥ 2 or ≤ 0.5 were set as the cut-off criteria for identifying DEGs. To predict the gene function and calculate the functional category distribution frequency, Gene Ontology (GO) analyses and enriched KEGG pathway were identified using KOBAS 2.0 server [68]. Hypergeometric test and Benjamin-Hochberg FDR controlling procedure were used to define the enrichment of each pathway (corrected *p*-value < 0.05).

### Real time qPCR validation of DEGs and rRNA genes

In this study, to elucidate the validity of the RNA-seq data, quantitative real-time PCR (qPCR) was performed for some selected DEGs, and normalized with the reference gene GAPDH. The information of primers is presented in Table S13. The same RNA samples for RNA-seq were used for qPCR. The PCR conditions are consisted of denaturing at 95˚C for 10 min, 40 cycles of denaturing at 95˚C for 15 s, annealing and extension at 60˚C for 1 min. PCR amplifications were performed in triplicate for each sample.

### UV cross-linking, immunoprecipitation, and sequencing (iRIP-seq)

Cells were irradiated on ice with type C (254 nm) UV at 400 mJ/cm^2^ for cross-linking. The cross-linked cells were lysed in ice-cold lysis buffer (10 mM HEPES, pH 7.0, 100 mM KCl, 5 mM MgCl2, 0.5% NP-40, 10 mM DTT) with 200 U/ml RNase inhibitor (Promega) and a protease inhibitor (Roche) on ice for 5 min. The mixture was then vibrated vigorously and centrifuged at 13,000 × g at 4 °C for 20 min to remove cell debris. The supernatant was pre-cleared with 100 μl of DynaBeads protein G (Life Technologies) at 4 °C for 30 min. The pre-cleared supernatant was incubated with DynaBeads protein G conjugated with monoclonal Flag antibody (Sigma) or Input at 4℃ for 30 min. The beads were washed 6 times with lysis buffer and then divided into two groups, one for RNA isolation from HMGB1-RNA complexes and another for the western blotting assay for HMGB1 immunoprecipitation. The HMGB1-bound RNAs were isolated from the immunoprecipitation of monoclonal Flag antibody (Sigma), followed by the preparation of the Illumina Truseq pair-end libraries. In brief, the collected RNAs were fragmented at 95 °C, followed by end repair and 5′ adaptor ligation. The reverse transcription was performed with RT primers harboring 3′ adaptor sequence and random hexamer. The generated cDNAs were PCR-amplified and the 200–500 bp products were purified. For high-throughput sequencing, the libraries were prepared according to the manufacturer's instructions and applied to Illumina HiSeq X Ten system for 150 nt paired-end sequencing (ABlife. Inc., Wuhan, China).

### iRIP-seq data analysis and RIP-qPCR

For iRIP-seq data, data processing method was the same to the RNA-seq data. After processing, we merged the two technical replicates and aligned the combined reads to the human-GRCH38 genome using TopHat2 [35] with 2 mismatches. After alignment, HMGB1 bound regions (peaks) were identified by Piranha [37] and ABLIRC [36], respectively. To investigate the multiple mapped reads in the IP libraries, the processed reads were subsequently searched against the Rfam database and human rDNA sequences using Bowtie [69].

In order to detect whether HMGB1 bound RNAs were significantly and specifically enriched in the HMGB1 immunoprecipitation, we used normal PCR as a confirmation for the IP RNAs. We used input RNA as a reference and performed RT-qPCR to determine the relative level of specific RNAs bound by HMGB1. Sequence-specific PCR primer pairs are shown in Table S13.

### Immunofluorescence

Cells were grown on six-well plate coverslips, fixed with 4% paraformaldehyde for 20 min, and blocked with 5% BSA, then incubated with anti-HMGB1 (ab79823, Abcam), anti-FLAG (66,008–3-Ig, Proteintech), or anti-Nucleolin (10,556–1-AP, Proteintech), after that washed for three times to incubate Aluor-488 labeled rabbit antibody against anti-HMGB1, Aluor-488 labeled mouse antibody against anti-FLAG and Aluor-647 labeled rabbit antibody against anti-Nucleolin, after washed for three times, finally incubated with DAPI for 5 min and then analyzed fluorescence signals were captured by pannoramic SCAN II instrument (3DHistech, Hungary).

### LFQ quantitative proteomics

The samples were subjected to treatment with ultra-sonication, and proteins concentration was determined by BCA kit. After peptide purification using SDB-RPS desalting column, all samples were analyzed on an UltiMate 3000 RSLCnano system coupled on-line with Q Exactive HF mass spectrometer through a Nanospray Flex ion source (Thermo) following user manual. Peptide samples were injected into a C18 Trap column (75 µm*2 cm, 3 µm particle size, 100 Å pore size, Thermo), and seperated in a reversed-phase C18 analytical column packed in-house with ReproSil-Pur C18-AQ resin (75 µm*25 cm, 1.9 µm particle size, 100 Å pore size). The MS was operated in DDA top20 mode with a full scan range of 350–1500 m/z. MS raw data were analyzed with MaxQuant (V1.6.6.0) using the Andromeda database search algorithm. Proteins with a fold change > 1.5 or < 1/1.5 and a students’ *t*-tests *P*-value < 0.05 were identified as differentially expressed proteins (DEPs) and used for functional annotation.

### Detection of methylation modification in ribosomal RNAs using RTL-P method

To check the 2’-O-methylation sites in the 28S rRNA, we applied RTL-P assay [70]. RT was performed in 20 μl reaction mixture containing 0.5 μg of total RNA, 1 μl specific RT primers and low (5 μM), middle (0.5 mM) or high (2.5 mM) concentration of dNTPs. PCR reaction was performed as follows: denaturing at 95˚C for 10 min, 40 cycles of denaturing at 95˚C for 15 s, annealing and extension at 60˚C for 1 min. RT-qPCR signal intensities were analyzed using Bio-Rad Quantity One software.

### Analysis of nascent translation

For nascent translation assays, overexpression-HMGB1 and control cells were incubated in HPG medium (medium supplemented with Methionine- and Cysteine-free DMEM, 1 mg/ml BSA instead of FBS) for 30 min, followed by HPG medium plus 50 μM for 45 min. After incubation, the cells were fixed with 3.7% formaldehyde for 15 min. HPG was detected by Click-iT™ HPG Alexa Fluor™ 488 Protein Synthesis Assay Kit (Thermos Fisher Scientific, C10428). All samples were analyzed on a Flow Cytometer. Relative HPG signal was measured and quantified by comparing with the none-HPG incubation cells fluorescence intensity.

### Supplementary Information

**Supplementary Material 1.**

**Supplementary Material 2.**

**Supplementary Material 3.**

**Supplementary Material 4.**

**Supplementary Material 5.**

**Supplementary Material 6.**

**Supplementary Material 7.**

**Supplementary Material 8.**

**Supplementary Material 9.**

**Supplementary Material 10.**

**Supplementary Material 11.**

**Supplementary Material 12.**

**Supplementary Material 13.**

**Supplementary Material 14.**

## Data Availability

The iRIP-seq and RNA-seq datasets have been deposited in the Gene Expression Omnibus (GEO) Database with accession number GSE120693.
