# Supplementary material for: HMGB1 prefers to interact with structural RNAs and regulates rRNA methylation modification and translation in HeLa cells
Source: BMC Genomics. 2024 Apr 5;25:345. doi: 10.1186/s12864-024-10204-6 (PMC10996203; doi:10.1186/s12864-024-10204-6)
Supplement: Supplementary file 1 — Supplementary Material 1. [file 12864_2024_10204_MOESM1_ESM.docx]

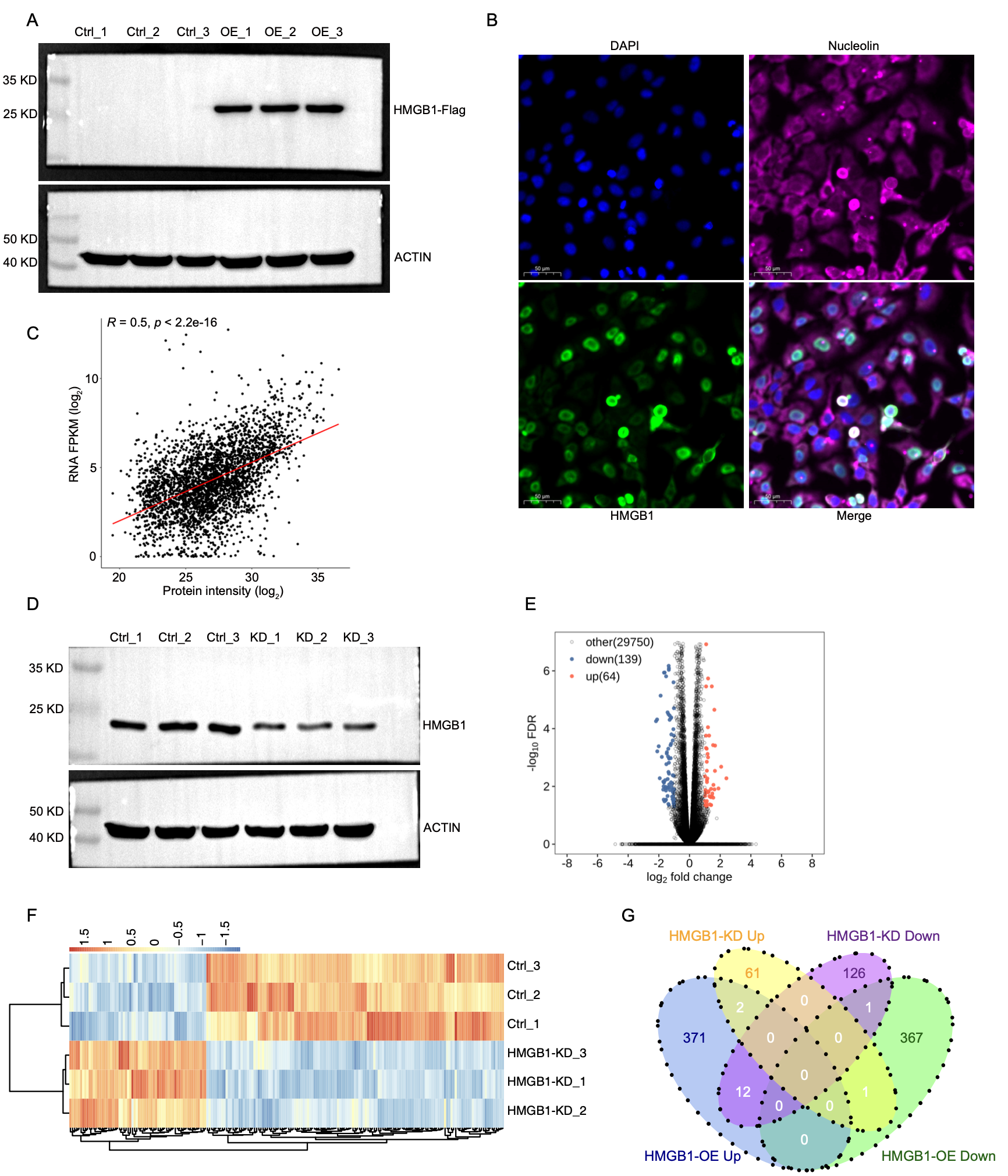


**Fig. S1 HMGB1-OE globally regulated the transcriptome profile in HeLa cells.** (A) The raw gels for WB result of HMGB1-OE and Ctrl samples. (B) Immune fluorescence experiment showing the subcellular localization of HMGB1 and Nucleolin in HeLa cells. (C) Sample correlation analysis for the detected protein level and RNA level in HMGB1-OE samples. (D) The raw gels for WB result of HMGB1-KD and Ctrl samples. (E) Detection of the HMGB1-KD regulated genes on the volcano plots, up DEGs (FC≥2, FDR< 0.05) are labeled red, whereas down DEGs (FC≤0.5, FDR< 0.05) are labeled blue. (F) Hierarchical clustering of DEGs in Ctrl and HMGB1-KD cells (Expression values are log2-transformed and the median-centered by each gene). (G) Venn diagram showing the overlapped DEGs between HMGB1-OE and HMGB1-KD groups.


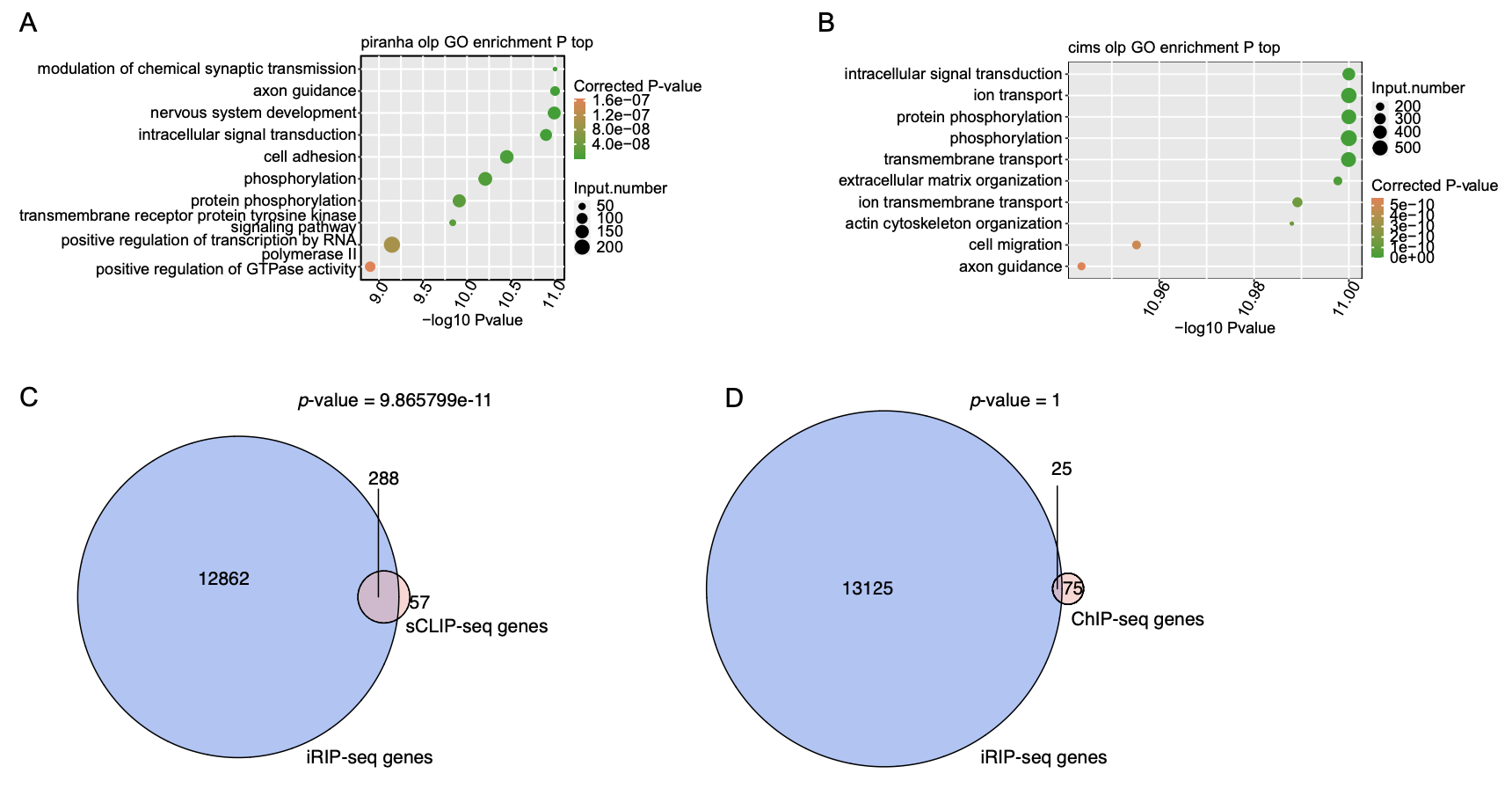


**Fig. S2. iRIP-seq of HMGB1 bound-RNAs in HeLa cells.** (A). The top 10 representative GO biological process terms of HMGB1-bound genes identified by Piranha algorithm. X-axis represents the base 10 logarithm of the enrichment p-value, y-axis represents the term of enriched GO pathways. (B) The top 10 representative GO biological process terms of HMGB1-bound genes identified by CIMS algorithm. X-axis represents the base 10 logarithm of the enrichment *p*-value, y-axis represents the term of enriched GO pathways. (C) Venn diagram showing the overlapped genes between iRIP-seq and sCLIP-seq identified genes. (D) Venn diagram showing the overlapped genes between iRIP-seq and ChIP-seq identified genes.


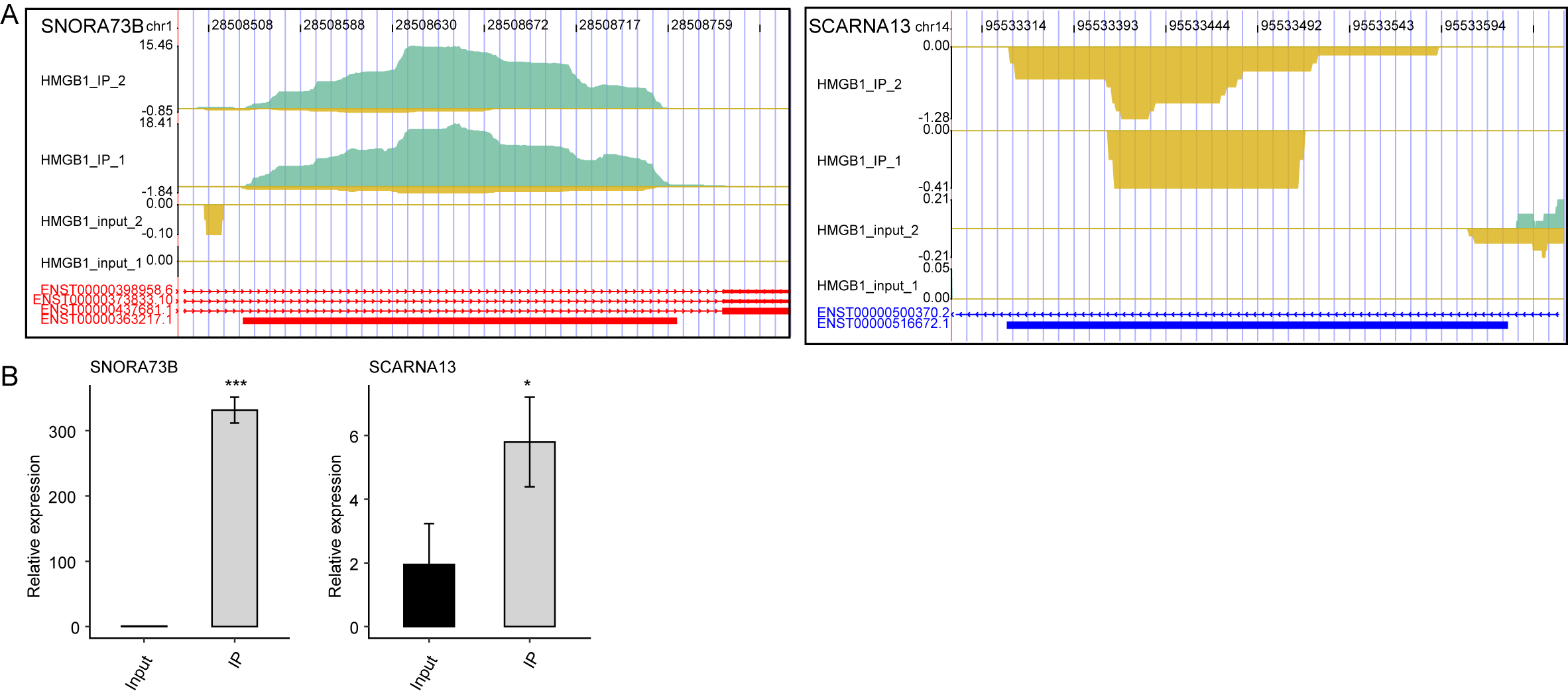


**Fig. S3 HMGB1 binds snoRNAs and scaRNAs with high affinity.** (A) The distribution of reads across the whole region in the snoRA73b and scaRNA13 genomic location and RIP-qPCR validation of HMGB1-bound. (B) Bar plot showing the uvRIP-qPCR results for SNORA73B and SCARNA13 that were bound by HMGB1. * *p*-value < 0.05; *** *p*-value < 0.001; Student’s *t*-test.
