# Supplementary material for: HMGB1 prefers to interact with structural RNAs and regulates rRNA methylation modification and translation in HeLa cells
Source: BMC Genomics. 2024 Apr 5;25:345. doi: 10.1186/s12864-024-10204-6 (PMC10996203; doi:10.1186/s12864-024-10204-6)
Supplement: Supplementary file 2 — Supplementary Material 2. [file 12864_2024_10204_MOESM2_ESM.docx]

Table S1. Summary of RNA-seq reads used in analysis

| Sample | HMGB1_1st | HMGB1_2nd | Ctrl_1st | Ctrl_2nd | Mean±SD |
| --- | --- | --- | --- | --- | --- |
| Raw reads | 65631292 | 69792042 | 73495554 | 70521544 | 69860108±3242503^a^ |
| Clean reads | 57157095 | 59080149 | 60633728 | 55557556 | 58107132±2216105 |
| Total mapped^b^ | 40048791(73.8%) | 46299552(81.97%) | 48718563(84.64%) | 44533704(84.95%) | 44900152.5±3660991 |
| Total Uniquely mapped^c^ | 34755504(86.78%) | 41682635(90.03%) | 44317736(90.97%) | 40495755(90.93%) | 40312907.5±4034558 |
| Splice reads^d^ | 15492331(44.58%) | 20117335(48.26%) | 22454154(50.67%) | 20613777(50.9%) | 19669399.25±2960589 |

a The mean and standard deviation across the 4 samples

b the percentage of paired-end reads that were mapped to the genome

c the percentage of unique reads mapping out of the total mapped reads

d the percentage of uniquely mapped reads that were mapped to splice site
